# Safety concerns to application of graphene compounds in pharmacy and medicine

**DOI:** 10.1186/2008-2231-22-23

**Published:** 2014-01-22

**Authors:** Mehdi Mogharabi, Mohammad Abdollahi, Mohammad Ali Faramarzi

**Affiliations:** 1 Department of Pharmaceutical Biotechnology, Faculty of Pharmacy & Biotechnology Research Center, Tehran University of Medical Sciences, Tehran 1417614411, Iran; 2 Department of Toxicology and Pharmacology, Faculty of Pharmacy and Pharmaceutical Sciences Research Center, Tehran University of Medical Sciences, Tehran 1417614411, Iran

**Keywords:** Graphene, Graphene oxide, Membrane, Reactive oxygen species, Safety, Toxicity

## Abstract

Graphene, the new allotrope of carbon is a single layer of monocrystalline graphite with sp^2^ hybridized carbon atoms. This compound has received worldwide attention due to its extraordinary physical and chemical properties. Duo to the widespread application of geraphenes, concerns are raising about its environmental safety or the safety protocols for handling and waste of graphene-based materials. The generation of reactive free radicals, adsorption of important biomolecules, and physical toxicity of graphene also matter. Hereby we criticize the concerns on the toxicity of graphenes to provide some perspective on the potential hazards of future development in graphene-based biomaterials.

## 

Graphene, a two-dimensional carbon sheet with single atom thickness has recently received significant interest due to its unique mechanical and electrical properties. Graphene is grown via chemical vapor deposition from carbon-containing gases on the surface of catalytic metals including Co, Pt, Pd, Ni, and Fe [1]. Geraphene derivatives possess high biocompatibility, physiological solubility and stability which make it efficient for biomedical applications such as biosensors, bioimaging, gene or drug delivery as well as tissue engineering and biocompatible scaffold for cell culture (Figure 1). Graphene derivatives have been extensively investigated for biosensing and detection of biomolecules such as oligonucleotide, thrombin, adenosine triphosphate (ATP), dopamine, and amino acids. Recently, graphene quantum dots (tiny nanoparticles with diameters in the range of 10-50 atoms) with ability of distribution in the body and also conjugation with biomolecules, have been widely investigated for medical imaging due to their tunable photoluminescence properties [2]. In addition, using functionalized graphene oxide for *in vivo* magnetic resonance imaging (MRI) demonstrated effective distribution of the nanocomposites after administration [3]. The mentioned potential applications of graphene indicate the high interest of researchers in this field. A brief overview of the number of grapheme-associated scholarly publications in the recent five years clearly supports our concern (Figure 2). Along with a rise in the number of patents related to the graphene, its industrial applications has been dramatically increased as several companies such as ACS material (USA), Anderlab technologies (India), Angstron materials (USA), CTI nanotechnologies (USA), and Durham graphene science (UK) started to produce graphenes involving single/multi-layer graphene, graphene oxide, graphene fluoride, 3D graphene foam, and graphene quantum dots [4]. However, commercial graphenes often supplied as fine dust with the potential health risks of inhalation. While scholars paid much attention to the safety concerns of nanomaterials [5,6], the potential toxic effects of graphene-based nanomaterials in environment and human health have never been studied well, yet [7]. Investigations on toxicity of graphene nanosheets in both Gram-positive and Gram-negative bacterial models have shown that graphene damages bacterial cell membranes through direct contact of the bacteria with extremely sharp edges of the nanowalls [8]. When tested in the respiratory tract, the graphene caused a milder toxicity on the epithelial cells and luminal macrophages in comparison to carbon nanotubes [9]. Particle size, particulate state, and oxygen content of graphene are key issues in its toxicity to human red blood and skin fibroblasts. It is known that graphene oxide induces cytotoxicity and genotoxicity in human lung fibroblasts through generation of reactive oxygen species and apoptosis [10]. The functional groups density on the surface of graphene oxide sheets play a key role in its cellular toxicity. In this regard, it is possible to reduce the toxicity by manipulating the surface functional groups or masking the oxygenated functional groups using a biocompatible polymer or manipulating the surface functional groups [11]. The effects of graphene oxide and polyvinylpyrrolidone modified graphene oxide on human immune cells have been investigated in vitro and showed that the latter has a lower immunogenicity than unadorned graphene oxide. Of course, the modification can increase the anti-phagocytosis ability of graphene oxide against macrophages with a significant improvement in biocompatibility of graphene oxide [12]. Graphene oxide is able to induce DNA cleavage which raises the concerns about potential toxicity of graphene oxide in human body [13]. The interactions between graphene sheets and various human plasma showed the affinity of low molecular weights proteins with graphene sheets surface resulting in formation of a complex between surface of nanoparticles and the proteins called corona [14]. The toxic effects of graphene on shoot and root growth, cell death, biomass, shape, and reactive oxygen species of several plants including cabbage, tomato, red spinach, and lettuce have been already investigated. The physiological and morphological analyses indicated that exposure to graphene inhibits the plant growth and biomass through overproduction of reactive free radicals [15]. The cytotoxic effects of graphene oxide prepared by different oxidative methods including Staudenmaier, Hofmann (concentrated nitric acid and KClO_3_ oxidant), Hummers (sodium nitrate for in-situ production of nitric acid in the presence of KMnO_4_), and Tour (concentrated phosphoric acid with KMnO_4_) were investigated in adherent lung epithelial cell. Different oxidative treatments resulted in production of graphene oxide with varying atomic C/O ratio which has an influence on toxicity profile of the graphene oxide [16]. Dimension, surface chemistry, and impurities as the most important properties of graphene derivatives directly influence their physiochemical and toxicity features. Recently, Bussy et al. [17] proposed strategies to enhance the overall safety of graphenes. Use of graphene sheets smaller than macrophages permits the immune system to remove extra particulates. Also, use of hydrophilic or modified degradable forms of graphene sheets have been proposed helpful. The cellular uptake mechanism and the intracellular metabolic pathway of graphene which are vital for *in vivo* applications of graphene should be studied in details [18]. The unique physical and chemical properties of graphene derivatives exhibit that there is still much need for scientific research and application development in smart targeted drug delivery, tissues engineering, disease diagnosis, and biosensors. Along with growing applications of graphene compounds in medicine, its toxicity profile must be completed and its safety concerns must be taken into account.

**Figure 1:**
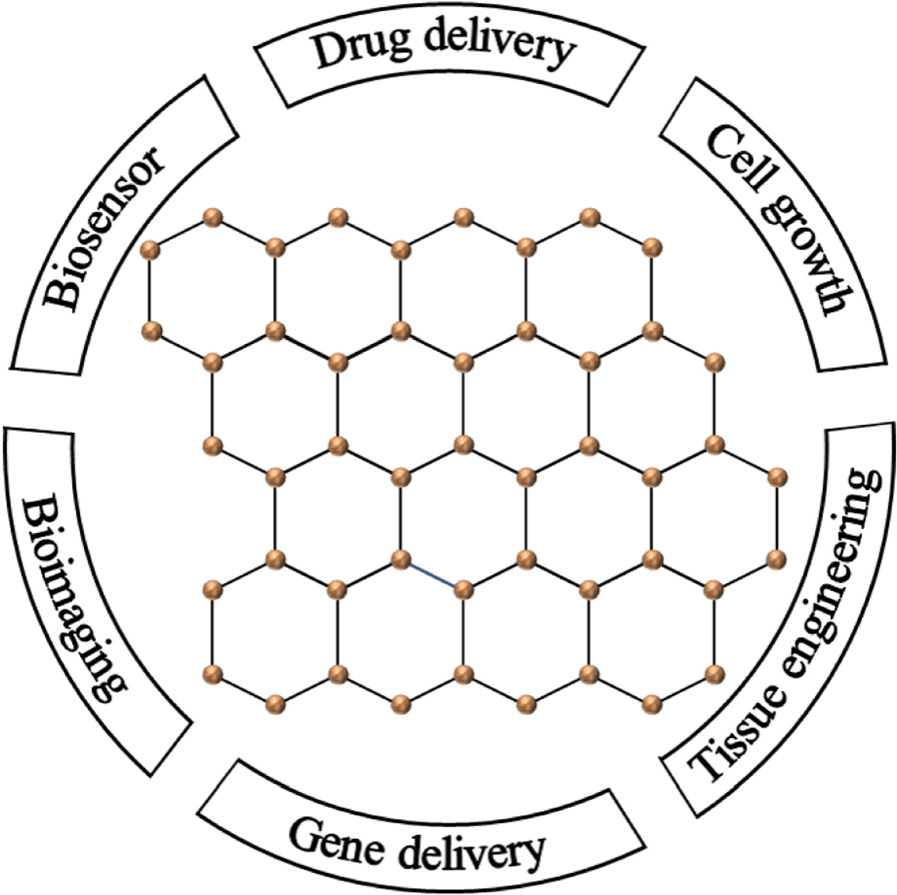
Various applications of graphene.

**Figure 2:**
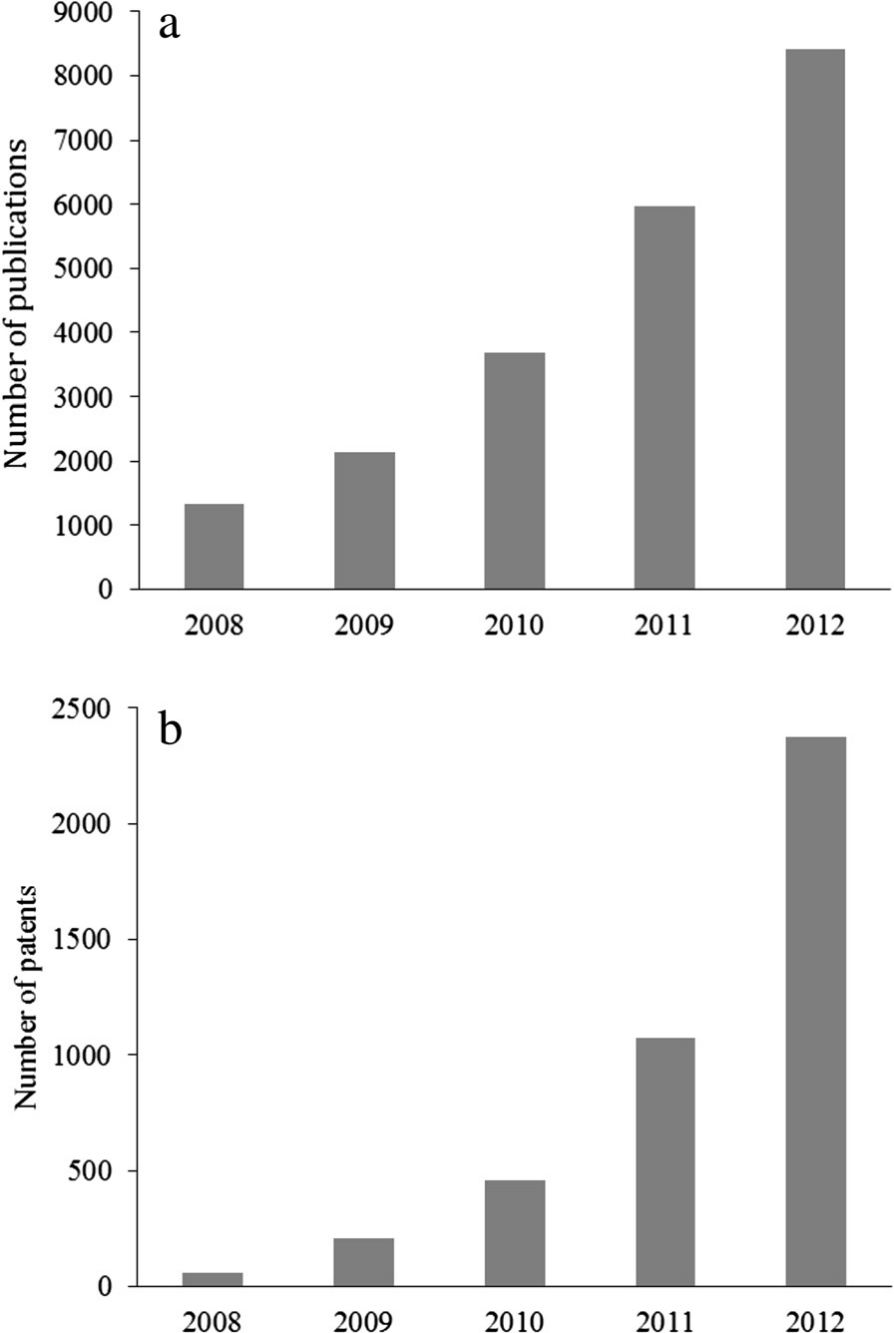
Number of graphene related publications (a), number of graphene related patents (b), based on data obtained from Scopus and European Patent Office (EPO) databases, respectively.

## Competing interests

The authors declared that they have no competing interests.

## Authors’ contributions

Authors contributed equally to the paper. Authors read and approved the final manuscript.
